# Insights on the functional interactions between miRNAs and copy number variations in the aging brain

**DOI:** 10.3389/fnmol.2013.00032

**Published:** 2013-10-02

**Authors:** Stephan Persengiev, Ivanela Kondova, Ronald Bontrop

**Affiliations:** Biomedical Primate Research CenterRijswijk, Netherlands

**Keywords:** miRNAs, CNV, brain aging, neurodegeneration, non-coding RNA

## Abstract

MicroRNAs (miRNAs) are regulatory genetic elements that coordinate the expression of thousands of genes and play important roles in brain aging and neurodegeneration. DNA polymorphisms affecting miRNA biogenesis, dosage, and gene targeting may represent potentially functional variants. The consequences of single nucleotide polymorphisms affecting miRNA function were previously demonstrated by both experimental and computational methods. However, little is known about how copy number variations (CNVs) influence miRNA metabolism and regulatory networks. We discuss potential mechanisms of CNVs-mediated effects on miRNA function and regulation that might have consequences for brain aging. We argue that CNVs, which potentially can alter miRNA expression, regulation or target gene recognition, are possible functional variants and should be considered high priority candidates in genotype–phenotype mapping studies of brain-related disorders.

## INTRODUCTION

The establishment of human cognitive abilities is a gradual process that takes place mostly in the period between birth and adulthood, although some developmental processes extend beyond this period (Sowell et al., 2004; Thompson et al., 2004; Zhan et al., 2013). During this time window, the brain undergoes dramatic molecular transformations, which are manifested both structurally and functionally (de Graaf-Peters and Hadders-Algra, 2006). Notably, shortly after the brain development is accomplished, the process of brain aging commences at early adulthood, which is revealed by the gradual decline of the brain ability to absorb and process the flow of information (Sowell et al., 2004; Peters et al., 2008; Salthouse, 2009; May, 2011; Zhan et al., 2013). However, more recent research has revealed that changes in brain circuits are not exclusively restricted to the early stages of brain development, and has supported the concept of continuous neuroplasticity throughout live (May, 2011; Taubert et al., 2012). Novel experience as a result environmental changes and new learning experience have been recognized as stimulating factors of brain function and underlying neuroanatomic networks. Experiments with animals have showed that mice living in active environment exhibited a reduced neuronal age-dependent degeneration and achieved a greater threshold for age-dependent deficits (Kempermann et al., 2002; Fryer et al., 2011).

The aging process is confronted by various neuroprotective mechanisms that are genetically programed and underlie the dynamics of the brain adaptive responses. The sole purpose of the multiple cellular and functional events that take place during brain aging is to maintain neural cells functionality and structural integrity. In cases where the neuroprotective mechanisms are overwhelmed by the accumulation of toxic products, the result is progressive neurodegeneration, as observed in Alzheimer’s disease (AD), cerebellar ataxias, and Parkinson’s disease (PD). The neuroprotective mechanisms can be augmented by dietary and behavioral modifications, but the genetic predisposition to accelerated aging is likely to be the main driving factor that triggers and maintains the advance of neurodegeneration.

## miRNA MACHINERY REACTION TO THE BRAIN AGING

Non-coding RNAs and microRNAs (miRNAs) in particular, play an essential role in the regulation of a number of cell processes, including cell proliferation, development, differentiation, stress responses, blast transformation, and apoptosis. The rapid accumulation of knowledge in the field of miRNA research has revealed its role in regulating gene expression at transcriptional and post-transcriptional levels. Meanwhile, the role of miRNAs in senescence remains poorly understood. miRNAs regulate several pathways associated with the aging mechanisms, and recent genome-wide analysis of miRNA expression revealed age-related changes in their expression level (Kosik, 2006; Krichevsky et al., 2006; Cogswell et al., 2008; Hebert and De Strooper, 2009). These data have underscored the significance of miRNA in brain aging and neurodegeneration.

MicroRNA can affect pathways involved in aging, and miRNA profiling has shown significant alterations in their expression level. Importantly, recent data have shown the significance of miRNA in brain aging and neurodegeneration (Kosik, 2006; Krichevsky et al., 2006; Cogswell et al., 2008; Hebert and De Strooper, 2009). The genome-wide expression analysis of miRNAs in aging individuals revealed a general decline in miRNA levels that was linked to potential loss of control of genes that regulate the cell cycle progression and cell differentiation programing (Noren Hooten et al., 2010). Nine miRNAs (miR-103, miR-107, miR-128, miR-130a, miR-155, miR-24, miR-221, miR-496, and miR-1538) were identified to be significantly lower in the peripheral blood mononuclear cells of old individuals as compared to the young subjects were identified in this study.

The ability of miRNAs to regulate oxidative stress and cell death is displayed in relationship to the growth harmone/insulin-like growth factor (GH/IGF) pathway and several AD-related oxidative damaging proteins (Nakasa et al., 2008; Stanczyk et al., 2008; Wang et al., 2008). Oxidative DNA damage may occur due to free reactive oxygen species (ROS) binding to nucleic acids and thus preventing transcription and causing DNA damage (Cooke et al., 2003). miR-210 and miR-373 inhibit the expression of key DNA repair proteins following hypoxic stress (Crosby et al., 2009). p53, a critical factor for maintaining the genome integrity, is activated by DNA oxidative damage, which is partially due to the miR-29-induced repression of negative regulators of p53, p85a, and CDC42 (Park et al., 2009).

Apoptosis is an extremely important signaling events influenced by miRNAs, particularly in the context of aging and age-related diseases. Several members of the miR-34 family participate in the p53 network, which induces apoptosis, cell cycle arrest, and senescence (Chang et al., 2007; He et al., 2007). It appears that activation of apoptosis – through internal or external stimuli, leads to repression of miRNAs that would otherwise silence genes involved in activating the apoptosis cascade. The reciprocal action, once an apoptotic cascade is activated, is the upregulation of miRNAs targeting proliferative or cell-survival genes (Wang, 2007). These results illustrate the complexity of miRNA interactions and their contribution to the regulation of programed cell death mechanisms.

MicroRNAs play a role in the control of brain metabolism and subsequently the dynamic of miRNA expression levels reflects the cellular responses to aging progression and deterioration of neuronal functionality. Several miRNAs are selectively expressed in brain tissues (Landgraf et al., 2007) and the inactivation of miRNA processing enzyme Dicer was found to lead to rapid degeneration of Purkinje cells (Schaefer et al., 2007). The global signature of miRNA expression in the adult brain appears to be species-specific, as shown by several comparative studies carried out on different species (Lee et al., 2000; Fraser et al., 2005; Berezikov et al., 2006). Selected miRNAs have been shown to be involved in AD, spinocerebellar ataxias, PD, and other neurodegenerative pathologies (Lukiw, 2007, 2012; Cogswell et al., 2008; Nelson et al., 2008; Persengiev et al., 2012b; Dimmeler and Nicotera, 2013). Genome-wide screens of miRNAs and ncRNAs in the aging brain found that miRNA expression is differentially regulated in the cortex and cerebellum of humans and non-human primates. This observation is likely to reflect the temporal functional status of neuronal activity in the cortex and cerebellum. Despite the observation for the lack of unifying specific miRNA pattern associated with the brain aging, the ontological analysis of targeted genes revealed that they represent a relatively conserved group (Persengiev et al., 2011). Importantly, miR-144 was identified to be the sole miRNA that was consistently upregulated in the aging chimp and human cerebellum and cortex (Persengiev et al., 2011, 2012a). The mechanism underlying the selective increase of miR-144 transcripts is unknown at this point, but indicates that miR-144 might play a coordinating role in the post-transcriptional suppression of specific genes in the aging brain. The mechanisms that govern miRNA expression during brain development and aging are highly structured and largely unknown. Complex gene expression patterns are regulated at several levels, including regulation by *cis*-acting *trans*-regulatory factors or regulation on the basis of epigenetic modifications such as gene methylation and histone modifications that depend on the genomic landscape. Thus, the adaptive responses of the brain cells during the aging process, which is reflected by brain phenotypic changes and the associated pathologies, will depend on either the physical presence or accessibility of multiple regulatory elements.

## COPY NUMBER VARIATIONS ASSOCIATED WITH miRNA GENES AND BRAIN ANOMALIES

Copy number variations (CNVs) in non-coding regions can have profound effects on human phenotype (Klopocki and Mundlos, 2011). CNVs most common outcome is altering the copy number of an entire gene that is predisposed to a dosage effect. In a different scenario, CNVs can result in position effects and cause long-distance effects as far as 1 Mb from the translocation breakpoints. CNVs have been associated with several neuropsychiatric disorders, such as autism, schizophrenia, and bipolar disorder (Cook and Scherer, 2008; Lee and Scherer, 2010). Furthermore, CNVs have been associated with PD and early onset AD, which support the possibility of the existence of CNVs-driven mechanism(s) in PD and AD pathogenesis (Toft and Ross, 2010; McNaughton et al., 2012).

Copy number variations have an impact on the miRNA-mediated post-transcription regulatory network as well. miRNAs preferentially regulate the centers of protein interaction and metabolic networks (Liang and Li, 2007; Baek et al., 2008) and CNVs of miRNA genes may fluctuate the dosage balance of signal transduction pathways, metabolic flux, or protein complexes (Veitia, 2004; Veitia et al., 2008), leading eventually to individuals of the same population or different populations having different susceptibility to diseases. Although a comprehensive investigation to evaluate the CNV-miRNAs health risks among human populations is still lacking, recent experimental studies have confirmed the role of CNV-causing dysregulation of miRNAs in disease occurrence (Volinia et al., 2010). High-frequency copy number abnormalities occur in miRNA-containing regions throughout the genome in a range of human diseases (Zhang et al., 2006; Guo et al., 2008; Rossi et al., 2008; Wong et al., 2008), which is associated with altered expression of multiple genes and pathways (Reddy et al., 2009; Whitman et al., 2010). Genome-wide association studies have confirmed such associations for dozens of protein-coding genes and showed that CNVs capture at least 18% of the total detected genetic variation in gene expression (Stranger et al., 2007). The expression of miRNA genes is modified by CNVs and there is a correlation between somatic CNV and the miRNA levels. Thus, the CNV of functionally relevant miRNAs can modulate or predispose to certain complex genetic diseases.

Copy number variations are segments of genomic DNA that are roughly 1 kb to 1 Mb in length that show variable numbers of copies in the genome due to deletions or duplications and may cause the impairment of neuronal structures. The co-localization of all miRNA loci with known CNV regions was analyzed by using bioinformatics tools (Marcinkowska et al., 2011). In total, 209 copy number variable miRNA genes (CNV-miRNAs) in CNV regions deposited in the Database of Genomic Variations (DGV) have been identified and validated. Eleven CNV-miRNAs in two sets of CNVs have been classified as highly polymorphic. The overall conclusions from this *in silico* study were that miRNA loci are underrepresented in highly polymorphic and well-validated CNV regions consistent with their essential biological functions. The potential importance and consequences of the miRNAs presence in detected CNV regions, however, has been recognized in several other studies, suggesting that rare CNV-miRNA variants might have significant functional impact (Morley and Montgomery, 2001; Sebat et al., 2004; McCarroll et al., 2008).

At this stage, little is known about CNV of miRNA genes that can cause reduced cognitive ability in normal individuals during aging. miRNA copy number change can cause aberrant miRNA expression and/or deregulation of their target genes in subjects with neurodegenerative disorders, intellectual disability, and congenital abnormalities. For instance, the potential role of CNVs in AD has been investigated and identified a number of genes overlapped by CNV calls (Heinzen et al., 2010; Swaminathan et al., 2012a,b). Case-control association revealed several loci containing CHRFAM7A, RELN, DOPEY2, CSMD1, HNRNPCL1, IMMP2L, SLC35F2, NRXN1, ERBB4, and HLA-DRA genes that are associated with AD. The NRX1 gene has been linked to AD, autism, and schizophrenia (Szatmari et al., 2007; Latella et al., 2009) and ERBB4 is likely to play a role in AD progression (Woo et al., 2010). Overall, there appears that gene duplications and deletions across AD cohorts might account for the differences in the individual susceptibility to the neurodegeneration progression.

Copy number variations were established to be a major contributor of the pathology of brain disorders, but almost all studies have focused on the protein-coding genes present in the CNV loci, while the impact of miRNAs present in these regions has been overlooked. In a more recent study the biological and functional significance of miRNAs present in CNV loci and their target genes has been addressed by using an array of computational tools (Vaishnavi et al., 2013). The study found that nearly 11% of the autism-associated CNV loci harbor miRNAs, most of which were not previously reported to be associated with autism. A systematic analysis of the CNV-miRNAs based on their interactions with the target genes enabled the authors to pinpoint 10 miRNAs, miR-590-3p, miR-944, miR-570, miR-34a, miR-124, miR-548f, miR-429, miR-200b, miR-195, and miR-497 as core factors. The newly identified autism-associated miRNAs were predicted to form a regulatory loop with transcription factors and their downstream target genes. In addition, miRNAs present in deleted and duplicated CNV loci may explain the difference in dosage of the crucial autism genes and can also affect core components of miRNA processing machinery through negative feedback loops. Interestingly, the most common genomic disorder in humans, the hemizygous deletion of a 1.5–3 Mb region of chromosome 22q11.2, which increases the risk of developing schizophrenia by approximately 25-fold includes *DGCR8* miRNA processing gene (Brzustowicz and Bassett, 2012). The exact mechanism by which this deletion increases risk is unknown, but the observation strongly suggests that altered miRNAs metabolism may be a factor in the pathogenesis of schizophrenia. Overall, the findings support a possible role of copy number change in miRNA expression and processing with consequences affecting cognition, brain disorders, and/or CNV-mediated developmental delay.

## EFFECTS OF COPY NUMBER VARIATIONS ON miRNA FUNCTION

Heritable information is transformed into cellular and organismal functions by the orderly expression of the entire set of genes in the genome. The complex process of gene expression regulation functions at several levels can be affected by structural alteration in the genomic architecture. Variations in the human genome occur on several levels. Originally, they were described as single-nucleotide changes within or outside of the coding sequence, or as microscopically visible alterations (CNVs) that affect parts of or even entire chromosomes. The effects include regulation in *cis* by promoters, enhancers, and repressors; regulation in *trans* by, e.g., transcription factors or miRNAs; or regulation on the basis of epigenetic modification such as DNA methylation. These genomic segmental differences reflect the dynamic nature of the genome and are believed to account for a large part of human phenotypic variability, including the predisposition to disease.

Selected genomic loci have been associated with non-coding pathogenic CNVs and their associated human disease phenotypes. CNVs were found to be distributed genome-wide that encompass non-coding sequences, thereby affecting the regulation of gene expression (Klopocki and Mundlos, 2011). More recently, a genome-wide scan identified 125 regions in which the same haplotypes are segregating in humans and chimpanzees, all with the exception of two encompassed non-coding regions (Leffler et al., 2013). In another study, a systematic search for DNA sequences missing in humans and present in chimpanzees, revealed that the identified sequences were almost exclusively from the non-coding regions of the genome (McLean et al., 2011). In addition, the study discovered that the absence of the penile bone in humans, which is present in chimpanzees, macaques, and mice, is due to the loss of a regulatory element that influences the expression of the androgen receptor gene. It is likely that these approaches will identify many more species-specific changes that relate to changes in phenotype.

Polymorphisms in miRNA genes can affect the expression of many downstream-regulated genes (Georges et al., 2007; Borel and Antonarakis, 2008). Single nucleotide mutations (SNPs) are most common form of polymorphism that affects the function of miRNAs, e.g., the structure of miRNA precursors, the efficiency of miRNA biogenesis and miRNA-target recognition. A series of *in silico* and experimental studies have revealed many SNPs located in different parts of miRNA genes (Duan et al., 2007; de Jong et al., 2013). The occurrence of SNPs in predominantly in the regions surrounding miRNA-coding elements, while sequences of mature miRNAs featured as the most conserved (Saunders et al., 2007). Functional analysis demonstrated that rare mutations naturally occurring within pre-miRNA sequences affect miRNA biogenesis and impair miRNA-mediated gene silencing (Duan et al., 2007; Sun et al., 2009). Recently, large genome-wide association study has demonstrated that SNPs located outside (>14 kb) of pre-miRNA sequences can modulate miRNA expression both as *cis*- and *trans*-regulators, as well (Borel et al., 2011). miRNA target sites are also conserved genetic elements and SNPs with potential to either disrupt or create new miRNA target sites are underrepresented in both experimentally validated and computationally predicted miRNA target sites (Chen and Rajewsky, 2006; Saunders et al., 2007; de Jong et al., 2013). Analysis of CNVs in the human and chimpanzee genomes demonstrates the potentially greater role of CNVs in evolutionary change than single base-pair sequence variation (Cheng et al., 2005). Comparisons of the human and chimpanzee genomes revealed that there are more than twice as many nucleotides involved in CNVs as there are in changes to individual nucleotides, 2.7% compared to 1.2%. Furthermore, the data revealed that while the majority of CNVs were shared between the human and chimpanzee genomes, approximately one-third of the CNVs observed in the human genome were unique and therefore acquired later in evolution. Additional studies have further revealed that CNVs are often linked to genetic diseases apparent in humans (Stankiewicz and Lupski, 2002). However, little is known about CNVs interactions with miRNAs.

**FIGURE 1:**
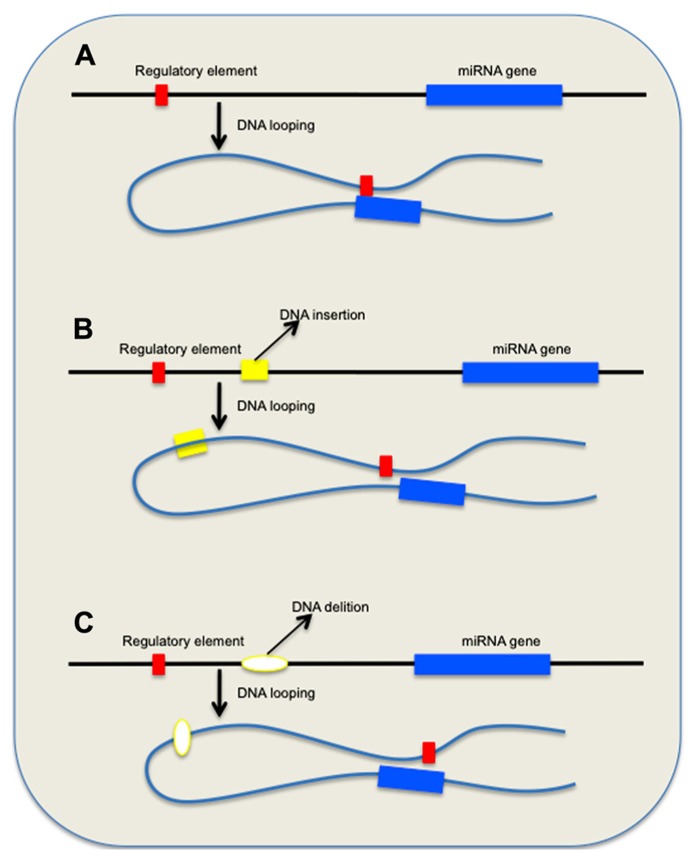
**Effect of genomic variations on long-range DNA interactions between remote regulatory elements and miRNA genes.** **(A)** DNA looping allows factors associated with distant regulatory element(s) to bind miRNA consensus sequences and control gene expression. **(B,C)** Scenarios that depict the effect of DNA insertions and deletions on the repositioning of the remote regulatory element(s) and eventual loss of physiological control mechanism.

Copy number variations have the propensity to alter the general organization of the chromatin in the affected chromosome regions that may have significant functional impact. Recent findings emphasized that nuclear architecture and chromatin organization play important role in the regulation of gene expression (Stankiewicz and Lupski, 2002), and that these components are essential epigenetic mechanisms for both the normal physiology as well as in the pathogenesis of a number of human maladies (Parada et al., 2004a). Portions of DNA, known as DNA loops, protrude from euchromatic portions of chromosomes, and the genes on these segments may localize to transcriptionally active chromatin centers that contain intergenic or intragenic miRNA genes (Osborne et al., 2004). Chromosome looping that enables remote segments of DNA from the same chromosome or from different chromosomes to interact and to modify the expression of distant genes presents a plausible mechanism that links the global misregulation of miRNA expression in AD and other neurodegenerative diseases to CNVs (**Figure 1**). As a consequence of CNV-induced chromatin reorganization, accessibility of miRNA binding elements within 3′ untranslated region (UTR) of target genes, miRNA promoters availability, as well as the expression of long ncRNAs that serve as sponges for miRNAs might be dramatically altered (Sanyal et al., 2012; Memczak et al., 2013). CNVs that are in close proximity of these loops may also trigger recombination and chromatin rearrangements (Parada et al., 2004b).

**FIGURE 2:**
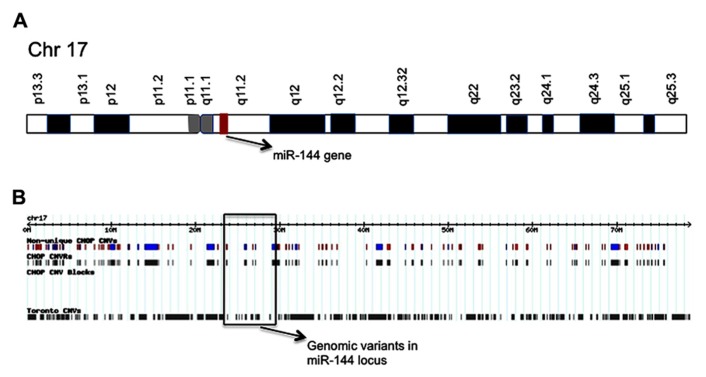
**Schematic of chromosome 17 showing the location of miR-144 gene (A); and distribution map of genetic variants identified in chromosome 17 (B).** The location of miR-144 gene is shown and CNVs in its respective genomic region are marked.

Interestingly, the aging-specific miR-144 is located on chromosome 17 in a region reported to be polymorphic, including several inversions and duplications, according to CNV database (**Figure 2**; **Table 1**). The significance of CNVs in the vicinity of miR-144 gene is unclear at this point, but long-range regulatory chromatin interactions play an important role in gene regulation. Both intrachromosomal and interchromosomal long-range associations have been demonstrated, and DNA binding factors have been implicated in the maintenance of these interactions (Cremer et al., 2000; Branco and Pombo, 2006). Several distant DNA segments may interact with a single gene and influence its expression pattern. Monoallelically expressed genes, most notably imprinted genes, are frequently found to be regulated by these long-range interactions. In support of this concept, FLT3-internal tandem duplications (ITDs) on chromosome 13, an adverse prognostic marker in specific aging individuals, were found to affect negatively the expression of GATA-3 transcription factor and miR-144 (Whitman et al., 2010). Members of GATA transcription factor family are believed to play a role in the control miR-144 transcription. GATA-4 transcription factor been reported to be critical regulator of miR-144 expression and is supposed to be the responsible gene for the congenital heart defects (CHDs) in the chromosomal 8p23 deletion syndrome, a complex malformation syndrome with clinical symptoms manifested by facial anomalies, microcephaly, mental retardation, and CHDs (Guida et al., 2010; Zhang et al., 2010). These findings emphasize the importance of studying the geography and architecture of the nucleus as an important factor in the regulation of miRNA expression.

**Table 1:** Genomic variations in the vicinity of miR-144 genomic location on chromosome 17 according to the Database for GenomicVariants.

Locus	Landmark	Variation type	Cytoband	Position (Mb)	Known genes in the locus
chr17:27013684-27014304	chr17:27,013,684..27,014,304	InDel	17q11.2	27.0
chr17:27107800-27123735	chr17:27,107,800..27,123,735	Copy number	17q11.2	27.1
	chr17:27,120,270..27,121,891	Copy number	17q11.2	27.1
chr17:27122880-27122983	chr17:27,122,880..27,122,983	InDel	17q11.2	27.1
chr17:27130078-27131878	chr17:27,130,078..27,131,878	Copy number	17q11.2	27.1
	chr17:27,130,682..27,131,776	Copy number	17q11.2	27.1
chr17:27130696-27131659	chr17:27,130,930..27,131,420	InDel	17q11.2	27.1
	chr17:27,130,738..27,131,656	InDel	17q11.2	27.1
	chr17:27,130,736..27,131,659	InDel	17q11.2	27.1
	chr17:27,130,696..27,131,638	InDel	17q11.2	27.1
chr17:27245834-27562095	chr17:27,459,989..27,461,612	Copy number	17q11.2	27.5	UTP6
	chr17:27,412,804..27,436,507	Copy number	17q11.2	27.4	SUZ12
	chr17:27,465,972..27,469,974	Copy number	17q11.2	27.5	LRRC37B
	chr17:27,245,834..27,562,095	Copy number	17q11.2	27.2	SH3GL1P1
	chr17:27,466,732..27,471,357	Copy number	17q11.2	27.5	ARGFXP2
	chr17:27,333,922..27,335,931	Copy number	17q11.2	27.3	RHOT1
chr17:27384860-27385274	chr17:27,384,860..27,385,274	InDel	17q11.2	27.4	LRRC37B
chr17:27460863-27461165	chr17:27,460,863..27,461,165	InDel	17q11.2	27.5
chr17:27614844-27619890	chr17:27,614,844..27,619,890	Copy number	17q11.2	27.6	RHBDL3
chr17:27621887-27622597	chr17:27,621,887..27,622,597	InDel	17q11.2	27.6	RHBDL3
chr17:27627845-27628095	chr17:27,627,845..27,628,095	InDel	17q11.2	27.6	RHBDL3
chr17:27633422-27634030	chr17:27,633,422..27,634,030	InDel	17q11.2	27.6	RHBDL3
chr17:27668824-27669757	chr17:27,668,824..27,669,757	InDel	17q11.2	27.7	RHBDL3
	chr17:27,669,594..27,669,594	InDel	17q11.2	27.7	
chr17:27788363-27788659	chr17:27,788,363..27,788,659	InDel	17q11.2	27.8
chr17:27837365-27838765	chr17:27,837,365..27,838,765	Copy number	17q11.2	27.8	CDK5R1
chr17:27917975-27917975	chr17:27,917,975..27,917,975	InDel	17q11.2	27.9	MYO1D
chr17:28279105-28280814	chr17:28,279,105..28,280,814	Copy number	17q11.2	28.3	TMEM98
chr17:28341799-28342792	chr17:28,341,799..28,342,792	InDel	17q11.2	28.3
chr17:28501812-28502008	chr17:28,501,828..28,502,008	InDel	17q11.2	28.5	ACCN1
	chr17:28,501,812..28,502,002	InDel	17q11.2	28.5
chr17:28620758-28620884	chr17:28,620,758..28,620,884	InDel	17q11.2	28.6	ACCN1
chr17:28630652-28631318	chr17:28,630,652..28,631,318	InDel	17q11.2	28.6	ACCN1
chr17:28643047-28645208	chr17:28,643,047..28,645,208	Copy number	17q11.2	28.6	ACCN1
chr17:28670843-28673962	chr17:28,670,843..28,673,962	Copy number	17q11.2	28.7	ACCN1
chr17:28708062-28708198	chr17:28,708,062..28,708,198	InDel	17q11.2	28.7	ACCN1
chr17:28779244-28781640	chr17:28,779,244..28,781,640	Copy number	17q11.2	28.8	ACCN1

*
miR-144 gene is encoded on the minus strand and contains two exons on Chr:17q11.2; 23,396,926-27,188,636 locus.*

## CONCLUSIONS AND OUTLOOK

The existing CNVs in the human genome cover approximately 360 Mb, or 12% of the human genome, as reported by the CNV Project database (). CNVs encompass more nucleotide content per genome than SNPs, underscoring CNVs’ significance to genetic diversity. A genome-wide map of CNVs shows that no region of the genome is exempt, and that between 6% and 19% of each individual’s chromosomes exhibit CNVs (Redon et al., 2006).

The mechanisms that operate during the progress of brain aging and associated neurodegenerative diseases are complex and their malfunction is rarely due to the failure of a few cell death or neuronal differentiation genes. Because susceptibility to premature aging and cognitive decline is a result of the malfunction of numerous genes, miRNAs dysregulation that inevitably would alter the expression of multiple genes might provide the basis for neuronal cell deterioration.

Multiple factors participate in the control of miRNA expression. Here, we discuss the emerging role of CNVs in miRNA regulation and the potential impacts on brain aging and neurodegeneration. Our simple notion is that the long-range interactions between DNA segments affected by CNVs might directly modify miRNA expression pattern, and as consequence miRNA-mediated inhibition of genes that are important for maintaining neuron homeostasis. We argue that CNVs-miRNA interactions are an important part of increased brain susceptibility to external and internal stress during the aging process. A more complete understanding of CNVs effect on the global nuclear geography and chromatin organization in the vicinity of miRNA-encoding regions will allow defining the chromosome regions that represent risk factors for the brain anomalies. Therefore, the challenge now is to annotate CNVs, which potentially can alter miRNA expression and determine whether they are functional variants and should be considered high-priority candidates in genotype–phenotype mapping studies of brain-related disorders.

## Conflict of Interest Statement

The authors declare that the research was conducted in the absence of any commercial or financial relationships that could be construed as a potential conflict of interest.
